# Vaccine Efficacy in Senescent Mice Challenged with Recombinant SARS-CoV Bearing Epidemic and Zoonotic Spike Variants 

**DOI:** 10.1371/journal.pmed.0030525

**Published:** 2006-12-26

**Authors:** Damon Deming, Timothy Sheahan, Mark Heise, Boyd Yount, Nancy Davis, Amy Sims, Mehul Suthar, Jack Harkema, Alan Whitmore, Raymond Pickles, Ande West, Eric Donaldson, Kristopher Curtis, Robert Johnston, Ralph Baric

**Affiliations:** 1 Department of Microbiology and Immunology, University of North Carolina at Chapel Hill, Chapel Hill, North Carolina, United States of America; 2 Carolina Vaccine Institute, University of North Carolina at Chapel Hill, Chapel Hill, North Carolina, United States of America; 3 Department of Genetics, University of North Carolina at Chapel Hill, Chapel Hill, North Carolina, United States of America; 4 Department of Epidemiology, University of North Carolina at Chapel Hill, Chapel Hill, North Carolina, United States of America; 5 Department of Pathobiology and Diagnostic Investigation, College of Veterinary Medicine, Michigan State University, East Lansing, Michigan, United States of America; 6 United States Army Medical Research Institute of Infectious Diseases, Fort Detrick, Frederick, Maryland, United States of America; The University of Hong Kong, People's Republic of China

## Abstract

**Background:**

In 2003, severe acute respiratory syndrome coronavirus (SARS-CoV) was identified as the etiological agent of severe acute respiratory syndrome, a disease characterized by severe pneumonia that sometimes results in death. SARS-CoV is a zoonotic virus that crossed the species barrier, most likely originating from bats or from other species including civets, raccoon dogs, domestic cats, swine, and rodents. A SARS-CoV vaccine should confer long-term protection, especially in vulnerable senescent populations, against both the 2003 epidemic strains and zoonotic strains that may yet emerge from animal reservoirs. We report the comprehensive investigation of SARS vaccine efficacy in young and senescent mice following homologous and heterologous challenge.

**Methods and Findings:**

Using Venezuelan equine encephalitis virus replicon particles (VRP) expressing the 2003 epidemic Urbani SARS-CoV strain spike (S) glycoprotein (VRP-S) or the nucleocapsid (N) protein from the same strain (VRP-N), we demonstrate that VRP-S, but not VRP-N vaccines provide complete short- and long-term protection against homologous strain challenge in young and senescent mice. To test VRP vaccine efficacy against a heterologous SARS-CoV, we used phylogenetic analyses, synthetic biology, and reverse genetics to construct a chimeric virus (icGDO3-S) encoding a synthetic S glycoprotein gene of the most genetically divergent human strain, GDO3, which clusters among the zoonotic SARS-CoV. icGD03-S replicated efficiently in human airway epithelial cells and in the lungs of young and senescent mice, and was highly resistant to neutralization with antisera directed against the Urbani strain. Although VRP-S vaccines provided complete short-term protection against heterologous icGD03-S challenge in young mice, only limited protection was seen in vaccinated senescent animals. VRP-N vaccines not only failed to protect from homologous or heterologous challenge, but resulted in enhanced immunopathology with eosinophilic infiltrates within the lungs of SARS-CoV–challenged mice. VRP-N–induced pathology presented at day 4, peaked around day 7, and persisted through day 14, and was likely mediated by cellular immune responses.

**Conclusions:**

This study identifies gaps and challenges in vaccine design for controlling future SARS-CoV zoonosis, especially in vulnerable elderly populations. The availability of a SARS-CoV virus bearing heterologous S glycoproteins provides a robust challenge inoculum for evaluating vaccine efficacy against zoonotic strains, the most likely source of future outbreaks.

## Abstract 2

Experiments in mice suggest challenges in vaccine design for controlling future SARS-CoV zoonosis, especially in vulnerable elderly populations.

## Editors' Summary

**Background.:**

Severe acute respiratory syndrome (SARS) is a flu-like illness and was first recognized in China in 2002, after which the disease rapidly spread around the world. SARS was associated with high death rates, much higher than those for flu. Around 10% of people recognized as being infected with SARS died, and the death rate approached 50% among elderly people. The virus causing SARS was identified as a member of the coronavirus family; it is generally thought that this virus “jumped” to humans from bats, which harbor related viruses. Although SARS was declared eradicated by the World Health Organization in May 2005, there is still the possibility that similar viruses will again cross the species barrier and infect humans, with potentially serious consequences. As a result, many groups are working to develop vaccines that will protect against SARS infection.

**Why Was This Study Done?:**

A SARS vaccine should be effective in people of all ages, including the elderly who are more likely to get seriously ill or die if they become infected. In addition, potential vaccines should protect against different variants of the virus, because there are different types of the virus that could potentially cross the species barrier from animals to humans. Of the different proteins that make up the SARS coronavirus, the spike glycoprotein is thought to elicit an immune response in humans that can protect against future infection. The researchers therefore examined vaccine candidates based on this particular protein (termed SARS-CoV S), as well as a second one called SARS-CoV N, in mice. Specifically, they tested whether the vaccines would protect against SARS infection in both young and older mice, and whether they would protect against infection by different strains of the SARS virus.

**What Did the Researchers Do and Find?:**

The researchers created vaccines based on SARS-CoV S and SARS-CoV N by taking the genes coding for those proteins and inserting them into another type of virus particle that acted as a delivery vehicle. They injected mice with these vaccines and then tested whether the mice generated an immune response against the specific SARS proteins, which they did. The next step was to work out whether mice injected with the vaccines would be protected against later infection with SARS-CoV. The researchers found that mice injected with vaccine based on SARS-CoV S were protected against later infection with a standard SARS-CoV strain, both in the short term (eight weeks after vaccination) and the long term (54 weeks after vaccination). However, the vaccine based on SARS-CoV N did not seem to result in protection, and, worryingly, caused pathological changes in the lungs of mice following virus challenge. To find out if their candidate vaccines would protect against different strains of SARS, the researchers made a synthetic test virus that contained a mixture of genetic material from different natural variants of the virus. This test virus was used to “challenge” mice that had been immunized with the two different vaccines. The researchers found that the vaccine based on SARS-CoV S protected against infection by the test virus when mice were vaccinated young, but it failed to efficiently protect when administered to older mice.

**What Do These Findings Mean?:**

The findings confirm others suggesting that vaccines based on the SARS-CoV S protein are more effective than those based on SARS-CoV N. They also suggest that the former can provide long-term protection in animals vaccinated young against closely related viruses. However, protection against more distantly related viruses remains a challenge, especially when vaccinating older animals. The differences seen between young and older mice suggest that older mice might provide a useful model for animal testing of candidate vaccines for diseases like SARS, flu, and West Nile virus that pose a particular threat to elderly people. Overall, these results provide useful lessons toward future SARS vaccine development in animals. The synthetic virus strain generated here, and others like it, are likely to be useful tools for such future studies.

**Additional Information.:**

Please access these Web sites via the online version of this summary at http://dx.doi.org/10.1371/journal.pmed.0030525.

• The World Health Organization provides guidance, archives, and other information resources on SARS

• Information from the US Centers for Disease Control on SARS

• Wikipedia (an internet encyclopedia anyone can edit) has an entry on SARS

• Collected resources from MedLinePlus about SARS

## Introduction

Severe acute respiratory syndrome coronavirus (SARS-CoV) infection results in severe acute respiratory disease, pneumonia, and sometimes death[1,2]. The disease was reported in Guangdong Province, China, in 2002 and spread to more than 30 nations within a few months. Disease severity was linked to age and other comorbidities, with mortality rates increasing with age and exceeding 50% in individuals over 65 y [3]. SARS-CoV is a zoonotic virus that crossed the species barrier, most likely originating from bats[4–6] or from other species including civets, raccoon dogs, domestic cats, swine, and rodents[7]. New zoonotic variants may emerge as evidenced by sporadic cases of human disease in late 2003 and early 2004, which arose from strains distinct from that of the epidemic [8]. In 2004, several laboratory-acquired infections were reported, including secondary spread resulting in fatal disease[9]. Given the significant health and economic impact, the development of an effective vaccine strategy that is protective against both epidemic and zoonotic SARS-CoV strains is highly desirable.

Attenuated and killed SARS-CoV, DNA, and viral vectored vaccines are being evaluated in a number of animal models including mouse, ferret, hamster, and primate [10–23], and have demonstrated that the SARS-CoV spike (S) glycoprotein is the principal component of protective immunity [15,24,25]. Although strong immune responses are elicited against both S glycoprotein and nucleocapsid (N) protein [10,15,26,27], passive transfer studies have illustrated that only anti-S antibody confers protection from SARS-CoV replication in the mouse model [16,17,28]. Vaccine development faces a series of potential concerns including reversion or recombination repair of attenuated vaccine strains, induction of immune-mediated enhancement of pathology, waning immune protection, lack of cross-protection for heterologous strains, and limited vaccine efficacy within senescent populations. Furthermore, immune enhancement has been demonstrated with another coronavirus, feline infectious peritonitis coronavirus [29], and more recently with a modified vaccinia vector expressing SARS-S that exacerbated hepatitis in ferrets while failing to protect from infection [30]. Notably, some antibodies against the epidemic Urbani strain increased the infectivity of lentiviruses pseudotyped with an animal SARS-S glycoprotein in an in vitro model, raising the specter of vaccine-mediated immune enhancement of disease following heterotypic challenge [31]. Another potential problem is that SARS vaccines might fail to induce antibodies that protect from infection with divergent strains of SARS-CoV. The S glycoprotein of SARS-CoV contains about 2%–20% amino acid variation between zoonotic and the 2003 epidemic strains [8,31], possibly limiting the effectiveness of monotypic SARS-S vaccines. Finally, studies measuring the duration of protective immunity or vaccine efficacy in animals greater than 4 mo post-boost have not yet been reported [17].

In this report, the efficacy of Venezuelan equine encephalitis virus replicon particle (VRP) vaccines expressing the Urbani SARS-CoV S glycoprotein (VRP-S) and N protein (VRP-N), either individually or in combination (VRP-S+N), are determined in young and senescent mouse models. We tested whether the senescent mouse model, which exhibits an age-related susceptibility to SARS-CoV similar to that seen in the human disease [32], will provide a sensitive measure of vaccine efficacy and reveal potential complications in SARS-CoV vaccine development for vulnerable elderly populations. We evaluated the duration of protective immunity following homologous and heterologous SARS virus challenge, examining the impact of waning immunity on long-term protection. Through the use of publicly available SARS-CoV sequence databases, bioinformatics approaches, synthetic biology, and reverse genetics, we constructed a viable heterologous challenge virus to test the ability of current vaccine regimens to protect against zoonotic strains; the likely source of future epidemics [8].

## Methods

### Viruses and Cells

The Urbani, Tor-2, recombinant Urbani (icSARS), and a recombinant chimeric virus encoding the S gene of GDO3 SARS-CoV (icGD03-S), strains were propagated on VeroE6 cells in Eagle's MEM supplemented with 10% fetal calf serum, kanamycin (0.25 μg/ml), and gentamycin (0.05 μg/ml) at 37 °C in a humidified CO_2_ incubator. For virus growth, cultures of VeroE6 cells were infected at a multiplicity of infection (MOI) of 1 for 1 h, the monolayer washed twice with 2 ml of PBS and overlaid with complete MEM. Virus samples were harvested at different times post-infection and titered by plaque assay. Plaques were visualized by neutral red staining and then counted.

Human nasal and tracheobronchial epithelial cells were obtained from airway specimens rejected from patients undergoing elective surgery under University of North Carolina (UNC) Institutional Review Board–approved protocols by the UNC Cystic Fibrosis (CF) Center Tissue Culture Core. Briefly, primary cells were expanded on plastic to generate passage 1 cells and plated at a density of 250,000 cells per well on permeable Transwell-Col (T-Col, 12-mm diameter; Corning [http://www.corning.com]) supports. Human airway epithelium (HAE) cultures were generated by provision of an air–liquid interface for 4–6 wk to form well-differentiated, polarized cultures that resemble in vivo pseudo-stratified mucosciliary epithelium, and infected with wild-type or recombinant SARS-CoV as previously described by our laboratory [33]. All virus work was performed in a biological safety cabinet (BSC cabinet) in a biosafety level three (BSL3) laboratory containing redundant HEPA-filtered exhaust fans. Personnel were double gloved and wore Tyvek suits with hoods supplied with HEPA-filtered air by a powered air-purifying respirator (PAPR).

### Construction and Isolation of the icGDO3-CoV Variant Virus

The GD03-S glycoprotein sequence has been reported. A synthetic DNA containing the 5′-most GD03 mutations was purchased (Blue Heron Biotechnology [http://www.blueheronbio.com]) and inserted into the SARS-E fragment. The plasmid clone (SARS-E GD03) was fully sequenced and shown to contain all of the appropriate mutations. The remaining GDO3 mutation was incorporated by PCR mutagenesis (5′ amplicon A: 5′-CTGTTTTCCCTGGGATCGC-3′; 3′ amplicon A: 5′-NNNNNNCACCTGCTTTTGGGCAACTCCAATGCC-3′; 5′ amplicon B: 5′-NNNNNNCACCTGCAGTTGCCCAAAATGTTCTCTATGAGAAC-3′; 3′ amplicon B: 5′- CATAAATTGGATCCATTGCTGG), followed by seamless ligation of the amplicons as previously described [34] into the SARS-F subclone. The final construct (SARS-F GD03) was fully sequenced and found to contain the appropriate set of GD03-S glycoprotein alleles.

The icGDO3-S was generated as previously described. Infectious clone fragment plasmid DNA was prepared in Escherichia coli (TOP-10, Invitrogen [http://www.invitrogen.com]), isolated, and purified (Qiagen [http://www.qiagen.com]). Infectious clone fragments B, C, D, and E were digested with BglI. Infectious clone fragments A and F were digested with EcoRI and NotI, respectively. Infectious clone fragments A and F were then dephosphorylated and then digested with BglI. Individual cDNA fragments were gel purified (Qiagen) and ligated (Roche [http://www.roche.com]) to form a full-length genomic cDNA and then chloroform extracted and EtOH precipitated. N cDNA and full-length viral genomic cDNA were then used as templates for in vitro transcription reactions (Ambion [http://www.ambion.com]). N and full-length viral genomic transcripts were then electroporated into Vero cells. Cell culture media containing virus was harvested 48 h post-electroporation. Virus was plaque purified and then passaged twice in Vero cells. The resultant stock was plaque titered and cyropreserved at −80 °C.

### Western Blot Analysis

Twelve hours post-infection, Urbani–, icSARS-CoV–, SARS-CoV–, Tor-2–, and icGD03-S–infected cells were washed in 1X PBS, lysed in buffer containing 20 mM Tris-HCl (pH 7.6), 150 mM NaCl, 0.5% deoxycholine, 1% Nonidet-p-40, 0.1% SDS, and post-nuclear supernatants added to an equal volume of 5 mM EDTA/0.9% SDS, resulting in a final SDS concentration of 0.5%. Samples were then heat inactivated for 30 min at 90 °C in the BL3 prior to removal. At BL2, samples were again heat inactivated for 30 min at 90 °C before use. Equivalent sample volumes were loaded onto 4% to 20% Criterion gradient gels (BioRad [http://www.bio-rad.com]) and then transferred to PVDF membrane (BioRad). Blots were probed with polyclonal mouse antisera directed against the Urbani-S glycoprotein diluted 1:200 or convalescent human sera 1128 diluted 1:400 and developed using electrogenerated chemiluminescence (ECL) reagents (Amersham Biosciences [http://www5.amershambiosciences.com]). Patient #1128 was infected during the second disease outbreak in Toronto, Canada.

### Plaque Reduction Neutralization Titer Assays

One hundred plaque-forming units (pfu) of either icSARS-CoV or icGDO3-S were treated with heat-inactivated serum diluted to final concentrations of 1:100, 1:200, 1:400, 1:800, or 1:1,600 and incubated at 37 °C for 30 min, and the resulting titer determined by plaque assay. Plaque numbers formed by virus treated with each dilution of sera from individual mice vaccinated with VRP-S or VRP-S+N were compared to the average number of plaques formed after treatment with a given dilution of sera from VRP expressing the influenza A HA protein (VRP-HA)- or PBS-vaccinated mice and expressed as the relative percentage. The dilution at which 80% of plaques were neutralized was determined for each VRP-S– or VRP-S+N–vaccinated animal.

### Mice

Female BALB/c mice (Charles River Laboratories [http://www.criver.com]) were anesthetized with a ketamine (1.3 mg/mouse) and xylazine (0.38 mg/mouse) mixture administered intraperitoneally with a 50-μl volume. Each mouse was intranasally (i.n.) inoculated with 50 μl of virus at a concentration of 2 × 10^6^ pfu/ml of virus. Four days post-infection, the right lung was removed and frozen at −70 °C for later plaque assay determination of viral titers. Half of the left lung was placed into Trizol Reagent (Invitrogen) for RNA extraction. The second half of the left lung was fixed in 4% PFA in PBS (pH 7.4) for at least 7 d prior to paraffin imbedding and sectioning for histopathological analysis. All mice were housed under sterile conditions, and sentinel mice were used to verify that the colony was mouse hepatitis virus (MHV) negative. Experimental protocols were reviewed and approved by the Institutional Animal Care and Use Committee at UNC Chapel Hill. Young mice refer to those challenged with SARS-CoV at ages equal or less than 5 mo old, whereas old or senescent mice are those animals with ages greater than 1 y at the time of challenge.

### Plaque Assay Titration of Virus from Lungs

Lungs were weighed and homogenized in four equivalent volumes of PBS to generate a 20% solution. The solution was centrifuged at 13,000 rpm on a tabletop centrifuge for 5 min, the clarified supernatant serially diluted in PBS, and 200-μl volumes of the dilutions placed onto monolayers of Vero cells in 60-mm dishes. Following a 1-h incubation at 37 °C, cells were overlaid with 1% agarose-containing medium. Two days later, plates were stained with neutral red and then plaques counted.

### VRP-S and VRP-N

The VRP constructs were made in two rounds of PCR, the first to generate two amplicons, and a second round of overlapping PCR to fuse them together. The fused DNA was digested with ApaI and AscI, and ligated into the similarly digested pVR21 plasmid. PCR reactions were performed with Expand Long Taq (Roche Molecular Biochemicals http://www.roche-applied-science.com) in 30 cycles of 94 °C for 30 s, 55 °C for 30 s, and extensions at 68 °C for 1 min. The first amplicon, which was used in the construction of both VRP-S and VRP-N, was generated with primers 5′nsp4Sw (5′-GATTGAGGCGGCTTTCGGCG) and 3′26S (5′-TTAATTAAGTCAATCGGCGCGCCCTTGGCGGACTAGACTATGTC) using pVR21 as template. The N-gene–specific amplicon was produced using primers V5′SARNg (5′-AGTCTAGTCCGCCAAGATGTCTGATAATGGACCCCAATC) and 3′SARSNg (5′-NNNNTTAATTAATTATGCCTGAGTTGAATCAGC) with SARS-F plasmid for template. The S-gene–containing amplicon was made with V5′SARSg (5′-AGTCTAGTCCGCCAAGATGTTTATTTTCTTATTATTTCTTACTCTCAC) and SARS3′Sg (5′-NNNNTTAATTAATTATGTGTAATGTAATTTGACACCC) using ligated SARS-E and -F fragments. The VRP-S and VRP-N cDNA templates were sequenced for verification and replicon particles produced as previously described [35]. Mice were vaccinated with 10^6^ infectious units (IU) of VRP in a 10-μl volume in the left rear footpad.

### Lung Histopathology

Lungs were fixed in 4% PFA in PBS for 7 d before being submitted to the Histopathology Core Facility (UNC, Chapel Hill) for paraffin imbedding, sectioning at 5-μm thickness, and hematoxylin and eosin staining. Approximately one-quarter of the total lungs were sectioned, with four to six sections mounted from cuts taken at five different depths within the paraffin-imbedded tissue. Lung pathology was scored in a blinded manner, in which six to ten sections per animal were evaluated and scored using the following scale. 1.0 to 2.0 = no to mild inflammation, 2.0 to 3.0 = mild to moderate inflammation, 3.0 to 4.0 = moderate to severe inflammation in less than half of the tissue section, and 4.0 to 5.0 = severe inflammation in more than half of the tissue section. The same sets of tissues were also evaluated qualitatively by a respiratory pathologist (author JH).

### In Situ Hybridization

The 5 μm–thick paraffin-embedded sections were probed with ^35^S UTP-labeled riboprobes complementary to the N gene of SARS-CoV (Urbani) or the HA gene of the A/PR8 strain of influenza as a negative control using previously described methods [36]. In brief, following treatment to prevent nonspecific probe binding, the tissues were incubated overnight with either probe at 5 × 10^4^ cpm/μl in hybridization buffer at 42 °C. The slides were then washed, dehydrated, and coated with NBT emulsion (Kodak [http://www.kodak.com]), and incubated at −80 °C. for 1 wk prior to development. Positive signal, as determined by silver grain deposition, was then evaluated.

### Enzyme-Linked Immunosorbent Assay

Antibody titers were determined by standard indirect enzyme-linked immunosorbent assay (ELISA). High-binding 96-well round-bottom plates (Corning [http://www.corning.com]) were coated with 10 μg/ml of SARS-S, SARS-N, or inactivated influenza A diluted in carbonate buffer containing 32 mM sodium carbonate, 68 mM sodium bicarbonate (pH 9.6) at 4 °C overnight. Mouse sera, diluted 1:100 in casein blocking buffer (Sigma [http://www.sigmaaldrich.com]), were added to wells in duplicate, and 2-fold serial dilutions were performed, followed by incubation for 2 h at 37 °C. Plates were then incubated for 1 h with goat anti-mouse IgG with alkaline phosphatase (AP) conjugate (Sigma), developed with *p*-nitrophenyl phosphate (*p*NPP; Sigma), and the optical density (OD) at 405 nm was measured (Bio-Rad Model 680 microplate reader). Log_10_ half-maximum ELISA titers were calculated with Sigmaplot (Systat [http://www.systat.com]) using the solution of the sigmoidal line of the plot of the log of the reciprocal dilutions of mouse sera and the resulting absorbances to determine the log of the reciprocal dilution at which an absorbance of 2.1 was achieved. Since very low amounts of antibody were being measured in the passive transfer experiment, log_10_ OD = 0.2 ELISA titers were calculated.

### Passive Sera Transfer

Mice were inoculated with 10^6^ IU of VRP-HA, VRP-S, or VRP-N at 7 wk of age, boosted 4 wk later, and terminally bled via cardiac puncture 3 wk post-boost. The sera of each group were pooled and 150 μl transferred by tail vein injection into mice at 7 or 43 wk of age. Mice receiving sera were bled and i.n. challenged with 10^5^ pfu of icSARS.

### Statistical Analysis

Unless otherwise noted, two-tailed Mann-Whitney tests were used for statistical comparisons. The Fisher exact tests were completed by comparing the number of animals positive for viral replication within the lungs of a group of animals vaccinated with VRP-S or VRP-S+N to that of the negative control group, VRP-HA or PBS. Values outside the limit of detection were assigned a value equal to the limit of detection for any analysis. The plus or minus (±) symbol is used to refer to standard deviation.

An amino acid multiple alignment was generated for the entire S gene of viral sequences representing early, middle, and late phases of the SARS epidemic in humans, as well as animal strains of SARS-CoV isolated from civets and racoon dogs found in Chinese live animal markets or housed on farms in China that supplied the markets. The sequences were aligned using ClustalX 1.83 with default settings [37]. A phylogenetic tree was generated using Bayesian inference as implemented in the program MrBayes v3.0b4 [38]. Briefly, the alignment was exported in the nexus format, the amino acid substitution model was set to JTT [39] using the lset command, and Markov chain Monte Carlo simulation [38] was used to approximate the posterior probabilities of trees with sampling conducted on four chains over 500,000 generations [40]. Trees were sampled every 100 generations, and the 5,001 trees collected were summarized with the sumt command set to a burnin of 1,000, which generated a consensus tree using the 50% majority rule [40]. The burnin value was determined using the sump command with an arbitrary burnin of 250, which demonstrated that stationarity occurred prior to the 100,000th generation, indicating that a burnin of 1,000 was appropriate for the sumt command [40].

## Results

### Venezuelan Equine Encephalitis Virus Replicon Particles Expressing SARS-CoV S and N

**Figure 1:**
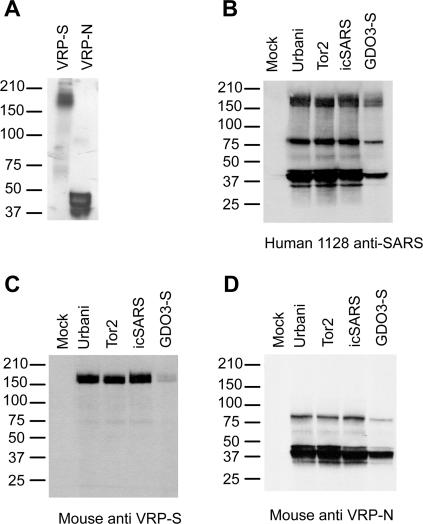
VRP Expression of SARS S and N and VRP-S Induction of Anti-SARS S Antibody (A) Western blot of cell lysates infected with VRP-S or VRP-N and probed with human serum collected from a convalescent SARS patient, 1128. (B–D) Western blot of lysates from cells infected with the SARS-CoV strains Urbani, Tor2, icSARS, or icGDO3-S and probed with human convalescent serum, 1128 (B), mouse anti–SARS-S serum from VRP-S–vaccinated animals (C), or mouse serum from a mouse vaccinated with VRP-N (D).

The SARS-CoV S glycoprotein gene and N protein gene were PCR cloned, sequence verified, and inserted into Venezuelan equine encephalitis VRPs. VRP-S and VRP-N constructs were packaged to give titers greater than 10^9^ IU per ml and shown to express antigenically relevant recombinant proteins. VRP-infected cell lysates were probed with antiserum 1128, derived from a convalescent human SARS patient. Western blot analysis of VRP-S–infected lysates revealed the expected S glycoprotein doublet of approximately 180–210 kDa, whereas that of VRP-N–infected lysates revealed a major product of less than 50 kDa, the expected sizes for SARS-S and -N, respectively (Figure 1A). VRP-S was inoculated into BALB/c mice and tested for its ability to induce antigen-specific antibody. Western blots were performed with Vero cell lysates infected with Urbani, SARS-CoV Tor-2, icSARS-CoV (the Urbani recombinant virus), and icGD03-S, a chimeric SARS-CoV expressing the S glycoprotein of the heterologous GDO3 strain. Blots were probed with anti–VRP-S mouse serum, 1128 human convalescent serum, or with anti–VRP-N mouse serum. The Western blots demonstrated that probing with human serum resolved bands corresponding to the major SARS antigens S (a doublet at ~180–210 kDa) and N (triplet at <50 kDa), as well as other unidentified SARS-CoV proteins (Figure 1B). Serum from mice vaccinated with VRP-S only identified SARS-S (Figure 1C), whereas serum from mice vaccinated with VRP-N recognized SARS-N in addition to another SARS-CoV protein that is probably a dimer of N (Figure 1D).

### VRP Vaccine Efficacy against icSARS-CoV Replication in the Mouse Model

**Figure 2:**
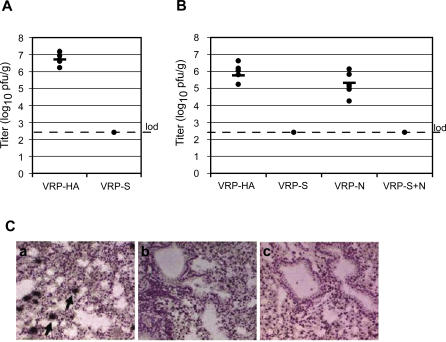
VRP-S Induces Short- and Long-Term Protection against icSARS-CoV Challenge icSARS titers are expressed as the log_10_ plaque-forming units per gram (pfu/g) of lung. Tissues were homogenized in PBS to form a 20% suspension and titered on Vero monolayers. The titers for individual mice are shown as a filled circle, and the mean titer for the group is represented by a solid bar. Limit of detection (lod) is 2.4 log_10_ pfu/g. (A) Lung titers of16-wk-old BALB/c mice harvested 2 d after being i.n. infected with 10^5^ PFU of icSARS-CoV (*n* = 6). (B) Lung titers of BALB/c mice vaccinated and boosted with 10^6^ infectious units (IU) of VRP expressing the influenza HA (VRP-HA), SARS-S glycoprotein (VRP-S), SARS-N protein (VRP-N), or a combination of VRP-S and VRP-N (VRP-S+N). Mice (*n* = 7 VRP-HA, *n* = 8 for other groups) were vaccinated at 5 wk of age, boosted 5 wk later, then i.n. challenged with 10^5^ pfu of icSARS-CoV 54-wk post-boost. Lungs were harvested 4 d later and titered. (C) Plaque assay results were confirmed by in situ hybridization to sectioned lungs of five mice from each vaccinated group with a radiolabeled riboprobe complementary to the SARS CoV N gene. In senescent mice challenged with the icSARS-CoV, representative lung sections from VRP-HA– (unpublished data) and VRP-N–vaccinated (a) animals exhibited extensive in situ signal (black arrows), whereas only one of five sections from VRP-S–vaccinated (b) and zero of five sections from VRP-S+N–vaccinated (c) mice exhibited SARS-CoV–specific signal above background levels.

As a general measure of vaccine efficacy (Table 1, experiment 1), six 4-wk-old BALB/c mice were vaccinated with either 10^5^ IU of VRP-S or VRP-HA, boosted 4 wk later with an equal amount of VRP, and then i.n. challenged with 10^5^ pfu of icSARS-CoV 8 wk post-boost. Consistent with other studies that made use of vectored SARS-S–expressing vaccines to induce protective responses [16,18,41,42], vaccination with VRP-S also prevented the replication of icSARS-CoV following challenge. No virus was detected by plaque assay (250 pfu/g limit of detection) in the lungs of VRP-S–vaccinated animals at 2 d post-infection, whereas the VRP-HA–vaccinated mouse lung had a mean titer of 6.7 ± 0.5 log_10_ pfu/g (Figure 2A). Vaccination with VRP-S demonstrated significant protection at the time of peak lung titer relative to VRP-HA–vaccinated control animals (*p* = 0.007, Fisher exact test).

**Table 1:**
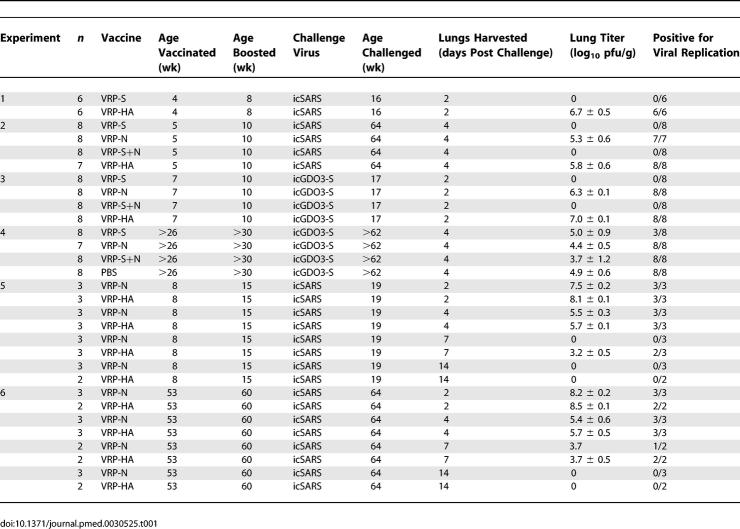
Summary of Vaccine Groups and Select Results for Mouse Experiments

A second vaccine experiment was completed to evaluate long-term VRP protection. Five-week-old BALB/c mice were vaccinated with 10^5^ IU of VRP-HA, VRP-S, VRP-N, or a combination of VRP-S and VRP-N (VRP-S+N), and boosted 5 wk later. Fifty-four weeks post-boost, mice were i.n. challenged with 10^5^ pfu of icSARS-CoV and lungs removed 4 d post-infection (summarized in Table 1, experiment 2). Although day 2 post-challenge demonstrates peak viral titers, day 4 was chosen to harvest lungs because it is the time at which the highest level of pathology is evident in senescent mice [32]. Titers in the lungs (Figure 2B) of animals vaccinated with VRP-S or the combination of VRP-S+N were below the limit of detection (250 pfu/g). In contrast, the lung titers of VRP-HA–vaccinated animals were 5.8 ± 0.6 log_10_ pfu/g, comparable to the VRP-N–vaccinated animal titers of 5.3 ± 0.6 log_10_ pfu/g (*p* = 0.2). These plaque assay results were confirmed by SARS-CoV–specific in situ hybridization on lung tissues from the infected mice (Figure 2C). Radiolabeled riboprobes complementary to the SARS-CoV N gene were hybridized to sectioned lungs of five mice from VRP-HA, VRP-N, VRP-S, or VRP-S+N vaccinated groups. Lung sections from VRP-HA (unpublished data) and VRP-N (Figure 2C, image a) vaccinated animals exhibited extensive in situ signal (arrows), whereas only one of five sections from VRP-S–vaccinated (Figure 2C, image b) and zero of five sections from VRP-S+N–vaccinated (Figure 2C, image c) mice exhibited SARS-CoV N-specific signal. Both VRP-S and the combination of VRP-S+N provided complete long-term protection against challenge with the homologous vaccine strain of SARS-CoV at 4 d post-infection (*p* < 0.001, Fisher exact test for both VRP-S- and VRP-S+N–vaccinated groups relative to VRP-HA).

### Protection against Heterologous Challenge

**Figure 3:**
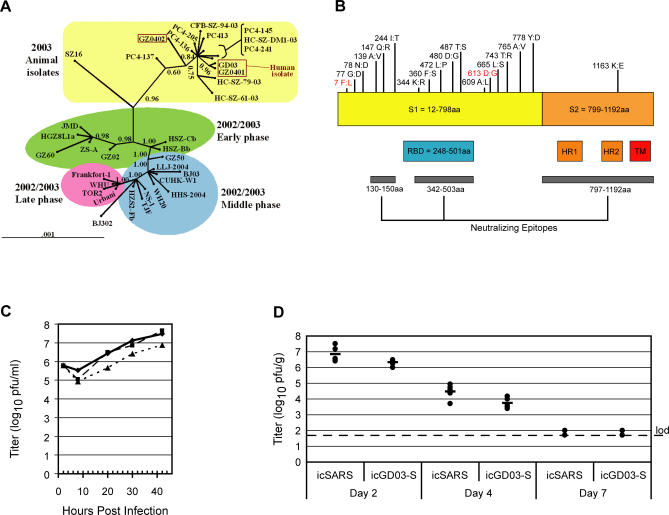
Synthetic Reconstruction of icGD03-S (A) Unrooted phylogenetic gene tree of 35 SARS isolates ranging from early, middle, and late phases of the 2002–2003 epidemic to 2003–2004 animal isolates. Branch confidence values are shown as posterior probabilities. The three human isolates that fall within the cluster otherwise isolated from animals (shown in boxes), GZ0402, GD03, and GZ0401, may represent infections in which a human acquired the virus from a Himalayan palm civet. (B) The GDO3-S glycoprotein. Amino acid changes unique to the GDO3-S with the GDO3-S amino acid listed on the left and the corresponding Urbani to the right. The GDO3-S amino acid changes are shown in relation to the S1 and S2 subunits, the receptor binding domain (RBD), heptad repeats one (HR1) and two (HR2), the transmembrane domain (TM), and known neutralizing epitopes. Two mutations that arose during tissue culture passage of the chimeric icGDO3-S are shown in red. (C) Growth curves of the Urbani strain of SARS-CoV (diamond, solid line), the recombinant Urbani icSARS (squares, dashed line), and the recombinant chimeric virus icGDO3-S (triangles, dotted line) in human airway epithelial cells. (D) Comparing growth of icSARS-CoV to icGDO3-S in the lungs of mice. Six-week-old female BALB/C mice were infected with icSARS-CoV or icGDO3-S (*n* = 5 per group). The individual titer of each mouse is represented by a filled circle, and the mean titer of the group is represented as a solid bar.

To perform cross-protection efficacy studies, it was necessary to construct a heterologous SARS-CoV. Selection of a likely candidate strain was made after Bayesian analysis of the SARS-CoV S glycoprotein, which demonstrated three main phylogenetic branches. Two of the branches include viruses isolated from animals, such as the palm civet and raccoon dog, and low pathogenic viruses sporadically isolated from humans, such as GDO3 and GZ0401.Viruses representing the 2003 early, middle, and late phases of the epidemic strains form the third branch in the SARS-S phylogenetic tree (Figure 3A). We resurrected the S glycoprotein of GDO3, a virus reported from a sporadic SARS case on December 22, 2003. Although GDO3 was not successfully isolated, its S glycoprotein was sequenced and reported. Compared to epidemic strains, GDO3 likely represented an independent introduction, was reported to be less pathogenic, and its S glycoprotein sequence is among the most divergent of all human strains [8]. The GDO3-S glycoprotein contains 17 amino acid changes relative to Urbani-S (Figure 3B), many of which map within neutralizing epitopes between amino acids 130–150 and 318–510, part of the receptor binding domain (RBD) [41,43–50]. Importantly, polyclonal antibody directed against the late-phase Urbani strain was less effective at neutralizing pseudotyped viruses bearing GDO3-S glycoproteins than those bearing Urbani-S [31]. The Urbani-S glycoprotein was removed from the SARS-CoV molecular clone, replaced with a synthetic cDNA encoding the GD03-S sequence, and used to generate recombinant virus [34]. Sequence analysis of plaque-purified icGDO3-S recombinant virus confirmed the presence of the GDO3-S glycoprotein and two additional changes in the S gene relative to Urbani-S (F7L and D613G), which likely arose as tissue-culture adaptations. The chimeric icGDO3-S, which only differs from Urbani SARS-CoV in its S glycoprotein, and wild-type icSARS-CoV recombinant viruses replicated in Vero cells to comparable titers that approached 10^7^ PFU/ml within 24 h (unpublished data) and their proteins were both detected in Western blots with human antiserum from convalescent patients (Figure 1B). Given the reduced amount of N present in the lysate of icGDO3-S–infected cells, the reduced intensity of the GDO3-S band probed with either anti–VRP-S mouse sera or the convalescent human serum is most likely due to the presence of lower GDO3-S protein rather than a marked difference in antibody specificity between GDO3-S and Urbani-S. icGD03-S replicated efficiently in HAE cells, although its maximum titer was approximately 1 log lower than that of icSARS or Urbani (Figure 3C). To compare the growth of icSARS and icGDO3-S in animals, 6-wk-old BALB/c mice were i.n. infected with 10^5^ pfu of either icSARS or icGDO3-S. At 2 d post-infection, icSARS-CoV mean lung titer was 6.8 ± 0.5 log_10_ pfu/g, whereas icGDO3-S titers were lower at 6.3 ± 0.2 log_10_ pfu/g (*p* = 0.04). The mean lung titer of icSARS-CoV–infected mice on day 4 was 4.5 ± 0.5 log_10_ pfu/g compared to icGDO3-S titers of 3.7 ± 0.3 (*p* = 0.04). By the seventh day, virus replication in the lungs of three of five mice infected with icSARS-CoV and four of five mice infected with icGDO3-S fell below the limit of detection (50 pfu/g). Average icSARS-CoV and icGDO3-S titers were similar on day 7 with 1.7 ± 0.1 log_10_ pfu/g and 1.8 ± 0.1 log_10_ pfu/g (*p* = 0.9), respectively (Figure 3D).

**Figure 4:**
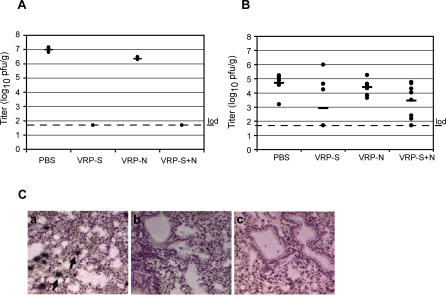
VRP-S Induces Short-Term Protection against icGDO3-S in Young and Partial Protection in Old Mice (A) Lung titers of BALB/c mice vaccinated and boosted with 10^6^ IU of VRP-S, VRP-N, a combination of VRP-S plus VRP-N (VRP-S+N), or mock vaccinated with PBS, then challenged with 10^5^ pfu of icGDO3-S challenge (*n* = 8 per group). Lungs were harvested 2 d post-challenge. (B) Lung titers of aged BALB/c mice vaccinated at greater than 26 wk of age, boosted 4 wk later, then challenged 12 wk post-boost with icGDO3-S (*n* = 7 VRP-N, *n* = 8 for other groups). Tissue was harvested 4 d post-challenge. (C) SARS CoV specific in situ signal (black arrows) was observed in the lungs of senescent mice that were vaccinated with PBS (unpublished data) or VRP-N (a) and challenged with icGDO3-S, although overall, the signal appeared to be less intense than that observed in the icSARS challenge animals (Figure 2C). Vaccination with VRP-S (b) or VRP-S+N (c) failed to induce complete protection from icGD03-S challenge, as sections from two of five S- and three of four S+N-vaccinated animals exhibited signal above that of uninfected controls.

To evaluate VRP protection against short-term heterologous challenge, groups of eight animals were primed at 7 wk of age with 10^6^ IU of VRP-S, VRP-N, VRP-S+N, or VRP-HA, boosted 3 wk later, and then challenged 7 wk post-boost with 10^5^ pfu of icGD03-S (summarized in Table 1, experiment 3). Lungs were harvested 2 d after challenge. VRP-S and VRP-S+N protected (*p* < 0.001, Fisher exact test for both VRP-S and VRP-S+N groups relative to VRP-HA) against heterologous icGD03-S recombinant virus replication (Figure 4A). Although high titers of virus were detected in VRP-N– and mock-vaccinated animals with mean titers of 6.3 ± 0.1 and 7.0 ± 0.1 log_10_ pfu/g, respectively, the VRP-N–vaccinated animals had a lower mean titer (*p* < 0.001).

SARS-CoV vaccines should confer protection to elderly subjects who face infection with a new variant of the virus. To model this scenario, we vaccinated 6-mo-old to 1-y-old BALB/c retired breeders with 10^6^ IU of VRP-S, VRP-N, VRP-S+N, or PBS, boosted them 4 wk later, and then challenged them 32 wk post-boost with 10^5^ pfu of icGDO3-S (summarized in Table 1, experiment 4). At 4 d post-infection, mean titers in the lungs of animals vaccinated with VRP-N and PBS were similar at 4.4 ± 0.5 and 4.7 ± 0.6 log_10_ pfu/g (*p* = 0.2), respectively (Figure 4B). VRP-S vaccination provided partial protection when compared to the PBS control group (*p* = 0.026, Fisher exact test), with the lungs of three of eight animals positive for viral replication at a mean titer of 2.9 ± 1.8 log_10_ pfu/g. All eight of the lungs harvested from the VRP-S+N–vaccinated animals were positive for viral replication, although a mean titer of 3.5 ± 1.2 log_10_ pfu/g was comparable to the mean titer for VRP-S–vaccinated mice (*p* = 0.4) and reduced relative to the PBS control (*p* = 0.02). The presence of SARS-CoV replication in the lungs of control and vaccinated animals was confirmed by in situ hybridization (Figure 4C). SARS-CoV N-specific riboprobe was hybridized to lung sections of mice from PBS-, VRP-N–, VRP-S–, and VRP-S+N–vaccinated groups (Figure 4C). All tested lung sections from PBS mocks (unpublished data) and VRP-N–vaccinated (Figure 4C, image a) animals exhibited in situ signal (arrows), although the signal did not appear to be as intense as that of the icSARS-CoV–infected animals (Figure 2C). Lungs of the VRP-S–vaccinated animals (Figure 4C, image b) had two of five slides exhibiting SARS-CoV–specific signal above background levels, whereas VRP-S+N (Figure 4C, image c) had three of four slides.

### Senescence and VRP-S Immune Responses

**Figure 5:**
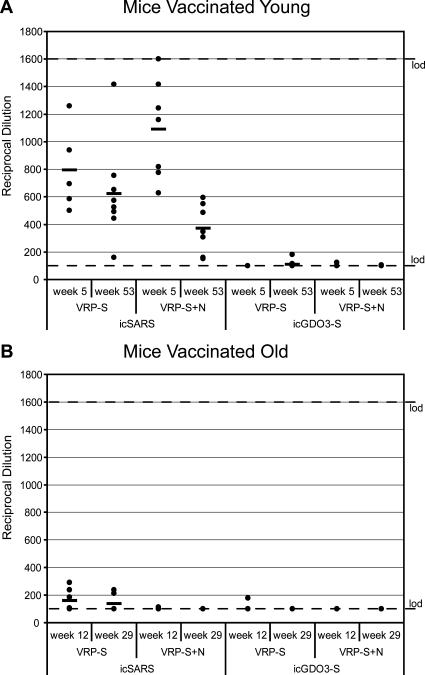
An 80% Plaque Reduction Neutralization Titers (PRNT_80_) for VRP-S and VRP-S+N Hyperimmune Serum (A) Mice vaccinated young: icSARS-CoV (left) and icGDO3-S (right) PRNT_80_ for VRP-S immune serum (experiment 2) collected at 5 wk post-boost (*n* = 5) and 53 wk post-boost (*n* = 8). (B) Mice vaccinated old: icSARS (left) and icGDO3-S (right) PRNT_80_ values for VRP-S and VRP-S+N immune serum (experiment 4) at 12 and 29 wk post-boost (*n* = 6 for icSARS; *n* = 5 for icGDO3-S). The PRNT_80_ values for individual animals are show as black circles, and the mean value is shown as a solid bar. The limits of detection (1:1,600 upper and 1:100 lower) are represented by horizontal dotted lines.

Because neutralizing antibody has been reported to confer protection from SARS-CoV replication within the lungs of mice [13,16,28], it was of interest to determine whether the VRP-S vaccine established high neutralizing antibody levels that persisted until challenge. Plaque reduction neutralization titer (PRNT) of serum samples harvested prior to vaccination showed no neutralization of icSARS-CoV; similar results were noted from serum collected from mice vaccinated with the negative controls, VRP-HA or PBS (unpublished data). The 80% PRNT values (PRNT_80_), the dilution of serum at which plaque numbers are reduced by 80% relative to virus treated with control sera, for VRP-S– and VRP-S+N–vaccinated animals at 5 and 53 wk post-boost against both icSARS-CoV and icGDO3-S were compared (Figure 5A; Table S1). The mean reciprocal dilutions for the PRNT_80_ of VRP-S and VRP-S+N against icSARS were measured at 796 ± 307 at 5 wk post-boost and 628 ± 363 at 53 wk. Sera from mice vaccinated with the combination of VRP-S+N had mean reciprocal PRNT_80_ of 1,091 ± 361 and 370 ± 179 at 5 and 53 wk, respectively. The initial neutralizing response in animals vaccinated with VRP-S and VRP-S+N was similar (*p* = 0.2 at 5 wk post-boost), and although there was not significant waning of the icSARS-neutralizing activity over the 48-wk period in the VRP-S–vaccinated animals (*p* = 0.3 Wilcoxin matched pairs signed-rank test), VRP-S+N serum was diminished by about 3-fold (*p* = 0.03 Wilcoxin matched pairs signed-rank test). All tested sera remained above the lower limit of detection (1:100) and were sufficient to prevent icSARS replication within the lungs of challenged animals (Figure 2B). The neutralizing activity of sera from the vaccinated animals was more effective against the vaccine strain than against heterologous icGDO3-S virus for both sera harvests and vaccine combinations. The reciprocal dilutions for the PRNT_80_ of the VRP-S samples at 5 wk post-boost were below the limit of detection, whereas two samples of the week 53 bleed were measured above the limit of detection with a mean value of 112 ± 28. The icGDO3-S PRNT_80_ measurements for VRP-S+N at weeks 5 and 53 post-boost were below the limit of detection with one exception for each time point: one mouse was measured to have a PRNT_80_ of 124 at 5 wk, for an average titer of 103 ± 8, and another a PRNT_80_ of 107 at 53 wk post-boost, an average of 101 ± 3.

Given that the VRP-S vaccine's ability to provide long-term protection was likely due to the strong SARS-CoV–neutralizing response induced in vaccinated mice, we measured the PRNT_80_ of the VRP-S immune sera from mice vaccinated when old (Table 1, experiment 4) to determine if the incomplete protection seen in that study could be linked to a reduced neutralizing antibody response in the senescent animals (Figure 5B; Table S1). Against the vaccine strain, the reciprocal dilutions of the mean PRNT_80_ were 170 ± 82 with two of six samples falling below the limit of detection for VRP-S mice at 12 wk and 142 ± 65 at 29 wk post-boost with four of six falling below the limit of detection. Sera from the VRP-S+N–inoculated mice had PRNT_80_ values falling near or below the limit of detection with only one measurable sample at 114, for an average of 103 ± 6 and no icSARS-neutralizing ability detected at week 29. Against icGDO3-S, the VRP-S PRNT_80_ values were below the limit of detection with the exception of a single VRP-S–vaccinated animal showing a neutralizing titer of 179 at 12 wk post-boost, for an average dilution of 116 ± 35 for the group. Sera harvested from these animals exhibited a marked reduction in neutralizing ability when compared to the response in animals vaccinated when young, even against the vaccine strain (*p* = 0.008 for VRP-S week 5 versus VPP-S week 12 post-boost; *p* = 0.006 VRP-S+N week 5 versus VRP-S+N week 12 post-boost). A strong anti–SARS-CoV neutralizing response was not induced by the VRP vaccines when administered to senescent mice.

**Figure 6:**
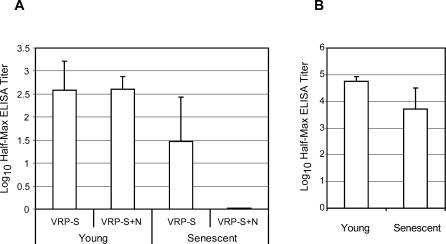
ELISA Titers for Anti-S and Anti-HA igG in Vaccinated Animals (A) Log_10_ half-maximum ELISA titers for anti-S IgG antibody in aged mice vaccinated with VRP-S or VRP-S+N when young (Table 1, experiment 2) or senescent (Table 1, experiment 4). Values represent mean values, and error bars indicate standard deviation. (B) Log_10_ half-maximum ELISA titers for anti-HA IgG antibody in aged mice vaccinated when young (experiment 2) or senescent (experiment 4).

ELISAs for total IgG specific for SARS-S and influenza-HA were performed to compare the VRP vaccines' ability to induce antibody to those antigens in mice vaccinated when young or senescent. ELISA for SARS-S was performed on sera collected prechallenge from the VRP-S– and VRP-S+N–vaccinated mice of experiments 2 and 4 (Figure 6A). Mice vaccinated young with VRP-S (experiment 2) had an average log_10_ half-maximum ELISA titer of 2.6 ± 0.6 at 53 wk post-boost, whereas that of the senescent animals was approximately a log lower at 1.5 ± 0.9 at 29 weeks post-boost (*p* = 0.007). The difference between animals of the two age groups was even more striking when anti-S IgG levels were compared in the VRP-S+N mice. The animals vaccinated young with VRP-S+N had an average titer of 2.6 ± 0.3, whereas the average for senescent animals was at the limit of detection of 0.02 log_10_ half-maximum ELISA titer. To verify that the reduced ability of the senescent animals to mount specific antibody responses was not limited to the SARS-S antigen; anti-HA IgG titers were compared in mice vaccinated with VRP-HA. The average anti-HA titer of animals vaccinated when young was 4.7 ± 0.2, whereas the titer of the vaccinated senescent mice was approximately one-tenth the size with a mean titer of 3.7 ± 0.8 (*p* < 0.001). The reduced ability of sera harvested from senescent animals to neutralize virus correlates to a general reduction in antigen-specific antibody production.

### Pathologic Findings in Mice

**Figure 7:**
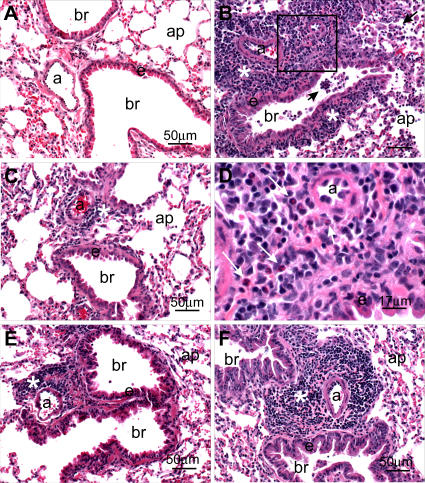
Pathogenic Findings Following Homologous Challenge Light photomicrographs of representative histologic lung sections (Table 1, experiment 2) taken from an untreated control mouse (A), a VRP-N–vaccinated mouse (B) and (D), a VRP-HA–vaccinated mouse (C), a VRP-S–vaccinated mouse (E), and a VRP-S– and VRP-N–treated mouse (F). No histopathology was evident in (A). A marked mixed inflammatory infiltrate composed mainly of mononuclear leukocytes (lymphocytes and plasma cells) and widely scattered eosinophils are evident in the perivascular and peribronchiolar interstitium (asterisk) in (B). Similar inflammatory cells are also present in bronchiolar (br) airways and alveolar airspaces along with enlarged and vacuolated alveolar macrophages (arrows). The box in (B) denotes the site of the light photomicrograph (D) that was taken at a higher magnification to better illustrate the lymphoplasmacytic inflammatory cell infiltrate with lesser numbers of eosinophils (arrows). Similar, but slightly less severe, perivascular inflammatory infiltrates (asterisk) are also present in (F), but without accompanying alveolitis. Minimal lymphoplasmacytic cell accumulations around the pulmonary arteriole (a) are evident in (C) and (E). All tissues were stained with hematoxylin and eosin. Bars denote the scale of the magnification. a, pulmonary arteriole; ap, alveolar parenchyma; br, bronchiolar lumen; e, surface epithelium of the bronchiole.

**Figure 8:**
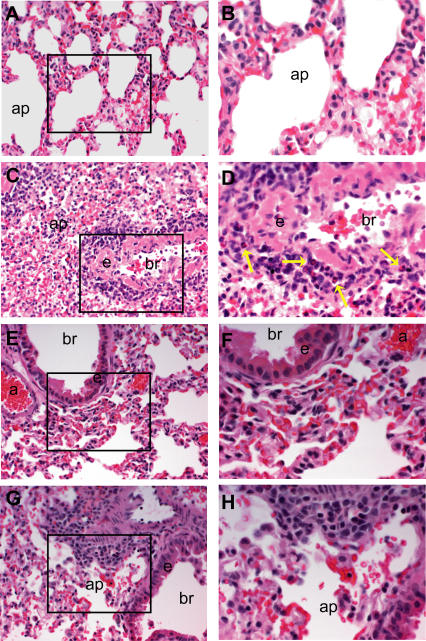
Pathogenic Findings Following Heterologous Challenge Light photomicrographs of representative histologic lung sections (Table 1, experiment 4) taken from a mock PBS–vaccinated mouse (A) and (B), a VRP-N–vaccinated mouse (C) and (D), a VRP-S–vaccinated mouse (E) and (F), or a VRP-S+N–vaccinated mouse (G) and (H). The boxes in (A), (C), (E), and (G) (200× magnification) denote the site of the light photomicrograph that was taken at a higher magnification (400×) to better illustrate the lymphoplasmacytic inflammatory cell infiltrates including eosinophils (yellow arrows). All tissues were stained with hematoxylin and eosin.

Lungs from the vaccinated senescent mice challenge studies (Table 1, experiments 2 and 4) were sectioned, hematoxylin and eosin stained, and analyzed for pathology. Though there was some animal-to-animal variation, in general only minor inflammatory changes were observed in the senescent control mice challenged with either icSARS or icGDO3-S (Figures 7C, 8A, and 8B). However, following SARS-CoV challenge in senescent mice, the N-vaccinated groups exhibited more marked bronchiolitis and alveolitis, as well as a conspicuous perivascular and peribronchiolar interstitial accumulation of numerous mononuclear leukocytes (mainly lymphocytes and plasma cells; i.e., lymphoplasmacytic cuffing) and increased numbers of widely scattered eosinophils (Figures 7B, 7C, 8C, and 8D). Upon SARS-CoV challenge, the animals vaccinated with S+N also exhibited a similar, but less severe, lymphoplasmacytic infiltration around pulmonary vessels and bronchiolar airways, although alveolitis and eosinophil infiltration were not a prominent feature in these animals (Figures 7F, 8G, and 8H). The lungs of animals vaccinated with VRP-S were similar to VRP-HA– or PBS mock-vaccinated animals with minimal lymphoplasmacytic cell accumulations (Figures 7E, 8E, and 8F). Therefore, not only did N vaccination fail to control SARS-CoV replication within the lungs, but N vaccination also resulted in an enhanced immunopathology in the lungs of the senescent animals upon viral challenge.

### Duration of N-Induced Pathology

In order to determine the kinetics of the VRP-N–associated immunopathology and whether this effect was age dependent, young and old VRP-N– or VRP-HA–vaccinated mice were challenged with icSARS and sacrificed on days 2, 4, 7, and 14 post-challenge. Young (8 wk of age) or senescent (53 wk of age) female BALB/c mice were vaccinated with 10^6^ IU of VRP-N or VRP-HA, boosted 7 wk later, then i.n. challenged with 10^5^ pfu of icSARS-CoV 4 wk post-boost. Lungs were harvested on days 2, 4, 7, and 14, titered, and processed for histology (Summarized in Table 1, experiments 5 and 6). In young mice, day 2 average lung titers were 7.5 ± 0.2 log_10_ pfu/g and 8.1 ± 0.1 log_10_ pfu/g for the VRP-N– and VRP-HA–vaccinated animals, respectively. Day 4 average titers were 5.5 ± 0.3 log_10_ pfu/g for VRP-N–vaccinated and 5.7 ± 0.1 log_10_ pfu/g for VRP-HA–vaccinated mice. SARS titers in the VRP-N–vaccinated mice lungs had dropped to the limit of detection by day 7, with two of the three lungs from VRP-HA–vaccinated mice showing measurable titers at a mean of 3.2 ± 0.3 log_10_ pfu/g. By day 14, virus was undetectable in either group. In senescent animals, the day 2 average lung titers were 8.2 ± 0.2 and 8.5 ± 0.1 log_10_ pfu/g for the VRP-N– and VRP-HA–vaccinated animals, respectively. By day 4, the mean titers had dropped to 5.4 ± 0.6 log_10_ pfu/g for VRP-N–vaccinated animals and 5.7 ± 0.5 log_10_ pfu/g for the VRP-HA controls. On day 7, one VRP-N–vaccinated mouse had a detectable titer of 3.7 log_10_ pfu/g, whereas the lungs of two VRP-HA–vaccinated mice were positive for replication with a titer of 3.7 ± 0.5 log_10_ pfu/g. No virus was detected in either group on day 14 post–challenge.

**Figure 9:**
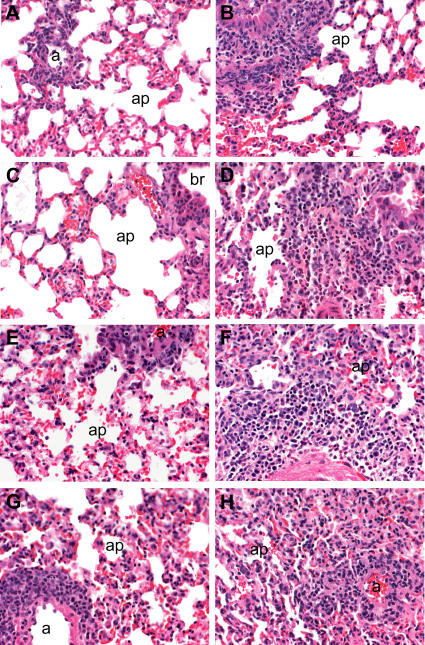
Kinetics of VRP-N–Associated Inflammation Light photomicrographs of lung sections taken from VRP-HA– and VRP-N–vaccinated mice harvested at days 2, 4, 7, and 14 post–icSARS-CoV challenge (Table 1, experiment 5). Representative lung sections (200× magnification) comparing pulmonary inflammation between VRP-HA–vaccinated (A), (C), (E), and (G) and VRP-N–vaccinated (B), (D), (F), and (H) mice. Enhanced inflammation was evident by day 2 (A) and (B) in some VRP-N–vaccinated animals relative to lung sections of VRP-HA–inoculated mice. By day 4 post-infection (C) and (D), increased inflammation in VRP-N–vaccinated animals was widely apparent and was maintained through days 7 (E) and (F) and 14 (G) and (H).

**Figure 10:**
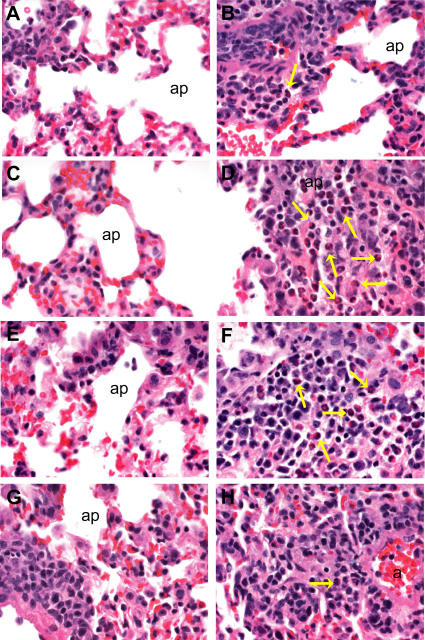
Identifying Eosinophils among Inflammatory Infiltrates The 400× magnification comparing eosinophil infiltration within the lung sections of VRP-HA–vaccinated (A), (C), (E), and (G) and VRP-N–vaccinated (B), (D), (F), and (H) mice (Table 1, Experiment 5). At day 2 post-infection (A) and (B), eosinophils are rarely evident in the lungs of either VRP-HA (A) or VRP-N (B) mice. Day 4 post-infection (C) and (D), extensive eosinophils (yellow arrows) are present within the lungs of VRP-N–vaccinated mice. Widespread eosinophils are seen at day 7 post-challenge in VRP-N–vaccinated (F), but not VRP-HA–vaccinated (E) mice. By day 14 (G) and (H), eosinophils are rarely found among inflammatory cells of VRP-N–vaccinated mice. An identical experiment in old animals was performed simultaneously (Table 1, experiment 6), showed results indistinguishable from those of young mice (unpublished data). All tissues were stained with hematoxylin and eosin.

Though enhanced inflammation was observed in a subset of the VRP-N–vaccinated animals at day 2 post–infection (Figure 9B), the inflammatory infiltrates were readily apparent in both young and senescent animals at day 4 post-infection (Figure 9D and unpublished data) and were maintained at days 7 and 14 post-infection (Figure 9F and 9H). As had been noted previously, VRP-HA– and VRP-N–vaccinated animals also differed by the presence of eosinophils (Figure 10). At day 2 post-infection, eosinophils were rarely seen in either VRP-HA– or VRP-N–vaccinated mice (Figure 10A and 10B, respectively). In VRP-N–vaccinated animals, but not HA–vaccinated animals, eosinophils were widespread on days 4 and 7 (Figure 10D and 10F), but had largely cleared from the lungs by day 14 (Figure 10H). There was no apparent difference in severity between the young and senescent animals, suggesting that the immune pathology was specific to pre-existing N immunity, but was not age dependent (unpublished data).

### Anti–SARS-N Antibody and Inflammation

A passive transfer of anti-N, anti-HA, or anti-S sera into naive mice was performed to determine if the increased inflammatory response could be attributed to N-specific antibody, and to confirm that protection in S-vaccinated animals was mediated by S-specific antibody. Hyperimmune sera against VRP-HA (5.26 log_10_ OD = 0.2 ELISA titer), VRP-N (5.6 log_10_ OD = 0.2 ELISA titer), or VRP-S (5.5 OD = 0.2 ELISA titer) were intravenously transferred in 150-μl volumes to groups of 8-wk-old or 43-wk-old naive BALB/c mice prior to challenge with 10^5^ pfu of icSARS. Prior to challenge, serum was collected from mice and antigen-specific IgG titered to verify successful transfer. Young mice receiving anti–VRP-HA (*n* = 4) had a mean ELISA serum titer of 2.8 ± 2.4 log_10_ OD = 0.2, whereas senescent mice (*n* = 4) had a 2.9 ± 1.8 log_10_ OD = 0.2 ELISA titer. The average serum titers for mice injected with anti–VRP-N were 2.7 ± 2.4 log_10_ OD = 0.2 ELISA titer in young (*n* = 4) and 2.3 ± 1.9 log_10_ OD = 0.2 ELISA titer in senescent animals (*n* = 4). The anti–VRP-S log_10_ OD = 0.2 ELISA titers were 3.1 ± 3.1 in young animals (*n* = 3) and 2.9 ± 2.3 in senescent mice (*n* = 3). Lungs were harvested 4 d post-challenge, virus titers determined, and processed for histological analysis. Virus titers in the young mice were 5.0 ± 0.1 log_10_ pfu/g in the anti–VRP-HA group, 5.2 ± 0.1 in the anti VRP-N group, and below the limit of detection (2.4 log_10_ pfu/g) in animals injected with the anti–VRP-S. Titers in the senescent mice were 5.3 ± 0.3 log_10_ pfu/g in the anti–VRP-HA group, 5.6 ± 0.6 in animals receiving the anti–VRP-N, and below the limit of detection in mice inoculated with anti–VRP-S. None of the mice displayed the enhanced inflammation noted in VRP-N–vaccinated animals (unpublished data), indicating that the observed immunopathology was not the result of antibody-dependent enhancement.

## Discussion

VRP vaccine vectors induce robust mucosal and cellular immune responses against a large number of foreign antigens [35,51] and were evaluated as candidate vaccines against homologous and zoonotic SARS-CoV challenge in young and senescent animals. Inoculation of mice with VRP-S induced antibody that recognized the epidemic SARS-CoV S glycoprotein as well as the S of a highly divergent strain, GDO3. The VRP-S vaccine induced long-term protection against challenge with the vaccine strain, complete short-term protection against icGDO3-S challenge, and partial protection against the divergent virus in the senescent mouse model. In contrast, vaccination with VRP-N failed to inhibit viral replication within the lungs of either young or senescent animals, resulted in enhanced immunopathology following viral challenge, and did not provide any measurable benefit when combined with VRP-S. The data suggest that vaccine regimens eliciting complete protection against antigenically heterologous forms of SARS-CoV in healthy individuals may not be sufficient for higher risk groups, including vulnerable elderly populations, and that there is a need for further testing of candidate vaccines that induce an anti-N response.

VRP-S vaccination generated a strong neutralizing antibody response (PRNT_80_ > 1:600) that persisted for over a year and provided complete protection against challenge with the vaccine strain. In humans, neutralization titers have been measured from 1:12 to 1:512 with a geometric mean titer of 1:61 [52]. In a study evaluating an inactivated virus vaccine, neutralizing antibody titers greater than 1:114 resulted in complete protection against challenge [21]. Mice inoculated with vesicular stomatitis virus vectors expressing SARS-S developed lower average neutralizing titers of 1:32, which were nevertheless protective against SARS-CoV infection for up to 4 mo after vaccination [17]. We followed animals for over 1 y after boost. To our knowledge, these are the first assays illustrating waning immune responses to a SARS-CoV candidate vaccine. On average, mice vaccinated when young with VRP-S did not show a significant reduction in neutralizing titers up to 53 wk post-boost, whereas mice vaccinated with the combination of VRP-S+N experienced about a 3-fold reduction over the same period of time. Although it is problematic to compare our neutralizing antibody titers to those induced by other SARS vaccines due to the use of different assays, we demonstrate protection from challenge with either vaccine or heterologous challenge virus strains in animals with an icSARS PRNT_80_ greater than 1:114, near the assay's limit of detection. Vaccines that induce robust neutralizing titers against the homologous strain will likely confer protection against zoonotic reintroductions, especially in younger populations.

As reported with other SARS-N–expressing DNA and vectored vaccines [15,24], VRP-N did not protect mice from SARS-CoV replication, and no benefit to vaccination with a cocktail of both VRP-S and VRP-N was observed, although an approximately half-log reduction in viral titers within the lungs of some VRP-N–vaccinated mice was occasionally observed. Although any reduction in SARS-CoV titer can be interpreted as a positive aspect of a potential vaccine, given the relationship between viral titer and SARS disease severity [53,54], the increased number of lymphocytic and eosinophilic inflammatory infiltrates, which are also characteristic of the immune pathology observed with respiratory syncytial virus (RSV) infection following vaccination with formalin-inactivated RSV [55,56], raises concerns that vaccination with N alone will not only fail to effectively protect against SARS-CoV replication, but may result in vaccine-enhanced pulmonary disease [57]. N-induced pathology has not been previously reported, probably because most studies examined young mice at 2–3 d post-infection, prior to the infiltration of inflammatory cells into the lung. VRP-N–induced pathology was clearly evident by day 4 and persisted for 1–2 wk following wild-type virus challenge, suggesting the potential for serious complications in lung physiology and function. This finding has particular significance for SARS-N and inactivated SARS-CoV vaccines currently under development that also induce anti-N antibody and T cell responses [12,19,21,26,58–61], because they may lead to adverse effects. Therefore, caution is merited with respect to the inclusion of SARS-CoV N protein in any vaccine formulation. The passive transfer of anti-N antibody did not contribute to inflammation and leads us to hypothesize that it is the activity of SARS-N–specific T cells in the absence of effective neutralizing anti–SARS-CoV antibody that mediates the adverse response. It is interesting that a T_h_2–skewed cytokine profile is a hallmark of the RSV vaccine-enhanced disease, which raises the possibility that the N-specific immune response is skewed in a similar manner [62].

SARS-CoV strain diversity was mostly confined to China where many human and animal isolates were not successfully cultured in vitro [8]. Consequently, most available experimental strains, like Urbani, are nearly identical and do not reflect natural diversity [63,64]. Recent advances in synthetic biology used to reconstruct extinct viruses, or specific genes of those viruses, de novo from their nucleotide sequences [65–67] provide the means for expanding the number of available SARS-CoV test strains. Using a comprehensive SARS-CoV genetic database [8,31], we resurrected the divergent GDO3-S glycoprotein in the Urbani genetic backbone. The icGD03-S recombinant virus was identical to the molecular clone except for the presence of two mutations in S that likely evolved after transfection of full-length RNA and virus passage in Vero cells, similar to the cell culture adaptations reported in S for other SARS-CoV strains isolated from human clinical specimens and passaged in vitro [68]. The icGDO3-S CoV's sequence divergence from Urbani, efficient in vitro replication in HAE and Vero cultures, and robust in vivo replication in the mouse model, make it an excellent heterologous challenge inoculum for vaccine studies. The GD03 RBD is also present in many zoonotic isolates described in civets and raccoon dogs, supporting its use as a zoonotic model strain [8]. Furthermore, the reduced replication in HAE cultures of icGDO3-S compared to Urbani-CoV is consistent with the reduced pathogenesis noted in the GDO3 human case [8,53,54].

Consistent with previous work comparing the susceptibility of pseudotyped lentiviruses bearing the S glycoproteins of various SARS strains to neutralization by anti-S (Urbani) IgG [31]**,** anti–VRP-S antibody demonstrated reduced neutralization of icGDO3-S relative to the vaccine strain. In spite of this, the VRP-S vaccine successfully provided short-term protection against the divergent virus. Vaccination of senescent animals produced significantly reduced antibody responses compared with younger mice, and when challenged with the heterologous icGDO3-S virus, protection was incomplete. However, any animal with a PRNT_80_ value above 1:114 against icSARS showed reduced viral replication within the lungs following challenge with either the vaccine or icGDO3-S strains. As noted for the homologous challenge studies, the combination of VRP-S+N did not enhance protection from heterologous challenge, but may actually have weakened it, with senescent animals showing even lower anti-S antibody responses and an even higher rate of viral replication, albeit with reduced titers, and increased lung pathology. One possible cause for vaccine failure is the emergence of an escape mutant in an environment of suboptimal neutralization. However, initial data comparing the neutralization susceptibility of viruses isolated from these mice to the challenge stock refute this conjecture (unpublished data). Incomplete protection by a vaccine in immunosenescent animals and humans is well documented and is more likely the result of an age-related compromise in one or more stages of the immune response to the vaccine. For instance, antibody responses in the immunosenescent tend to offer less protection with limited switching to secondary isotypes, lower antibody levels in general, and production of antibody with lower affinities [69–74]. Although we have not tested single-vaccine dose regimens, previous studies have demonstrated that these are efficacious against SARS-CoV challenge in young animals [17]. Given the low antibody titers following boost in senescent populations, single-vaccine dose formulations will likely prove ineffective. Rather, improving the VRP-S efficacy in older vaccinees may require additional vaccine boosts, the use of adjuvants, or other additional therapies [75]. Another likely contributing factor to vaccine failure in older animals was the resistance of icGDO3-S to neutralization relative to the vaccine strain, icSARS-CoV. At least three neutralizing sites have been identified in the SARS-CoV S glycoprotein, two of which map at the N-terminus and in the RBD of the S glycoprotein, and one to a weak third site near the carboxy-terminus of S. Given that most of the GD03 mutations map in and around the N-terminus and RBD in S1 [31], it is possible that either one or both of these critical epitopes are significantly different in icGD03-S, and likely explains the resistance to neutralization with antisera against Urbani-S. These data suggest that robust neutralizing titers should be induced by candidate vaccines to provide long-term protection from SARS-CoV infection, especially in the vulnerable senescent population and against heterologous strains.

Earlier work had indicated that antibodies to the Urbani strain of SARS-CoV enhanced the in vitro infectivity of pseudotyped viruses bearing the S glycoprotein of zoonotic strains, primarily with strains SZ16 and SZ3, and raised the specter of S-vaccine–induced complications with newly emergent strains [31]. In contrast, it was shown that monoclonal, but not polyclonal, antibodies that neutralized the epidemic strain may enhance the infectivity of pseudotyped viruses bearing GD03-S glycoproteins, although the enhanced infection was marginal at best [31]. Our research with antibody directed against Urbani-S indicated that the polyclonal antibody neutralized icGD03-S on Vero cells, although less efficiently than the vaccine strain, which is consistent with the previous report [31]. Moreover, in the young and senescent mouse models, VRP-S–vaccinated animals challenged with homologous or heterologous icGD03-S recombinant viruses did not display vaccine-mediated enhancement of virus replication or enhanced pathology. Because VRP-S vaccines induce broad neutralizing antibody responses that likely target multiple epitopes across the S glycoprotein, it is possible that the noted enhancement of infectivity with monoclonal antibodies is nullified. Indeed, recent work showed that antibody specific for the RBD of Tor-2–S, GDO3–S, and SZ3–S glycoproteins did not reproduce enhanced infectivity in pseudotyped viruses bearing SZ3-S and identified conserved epitopes that allowed all three strains to be effectively neutralized, raising hope that a single vaccine could be effective against widely divergent strains of SARS-CoV [76]. Clearly, additional studies are needed with more heterologous strains in alternative animal models before the possibility of vaccine-induced enhancement of infection and pathology can be discounted.

To our knowledge, this study is the first to demonstrate that a SARS-CoV vaccine conferred long-term protection into the period in which a host is most susceptible to SARS-CoV pathology: senescence. Furthermore, the VRP-S vaccination of young animals protected against challenge with a divergent strain of SARS-CoV, indicating that current vaccines may also provide protection from many zoonotic strains that might emerge in the future. Such cross protection has been observed among other vaccines, such as HA formulations for influenza virus [77] and VRP vaccines against norovirus [78]. Inducing robust immune responses in older animals is more challenging, but VRP-S vectors provided some protection from icGDO3-S challenge, and did so without the enhanced pathology induced by the VRP-N vaccine.

Because human infections have not been reported since 2004, animal models are essential for the development of SARS-CoV vaccines. The young mouse model provides readily available animals on a homogenous genetic background and efficient replication within the respiratory tract. The senescent mouse model adds the benefit of enhanced pathogenesis and mimicks the age-related susceptibility seen in humans [32]. Although aging decreases B and T cell immunity and innate immune function in humans and mice, characterization of these immune deficiencies is incomplete in both species. SARS-CoV infection in senescent mice provides a key model to evaluate the mechanisms by which aging deters immune responsiveness to highly pathogenic emerging viruses like SARS-CoV and influenza virus, and develop key intervention approaches to enhance vaccine efficacy in the elderly. The expense and limited availability of other senescent species makes the mouse model invaluable.

Important caveats must be considered while evaluating this work. Murine models of SARS disease have limitations. The disease progression in mice is faster than in humans, rodents and humans do not share the same symptoms, and virus infection is less severe; limitations that are also evident in the hamster, primate, and ferret models [79]. These shortcomings necessitate that vaccine candidates be tested in other animal systems and underscore the critical need for the development of highly pathogenic challenge models for vaccine and therapeutic testing. This report does not provide a mechanism for the VRP-N–induced pathology nor provide solutions for minimizing potential risks associated with it. Although the passive transfer of anti–SARS-N serum did not reproduce the inflammation seen in VRP-N–vaccinated animals, these results must be interpreted with caution because the anti-N antibody levels in the recipient mice were lower than those of mice directly immunized by VRP-N.

The data presented in this manuscript do reveal critical needs and potential complications in vaccine design, laying the foundation for continuing and future studies to improve the quality, safety, and efficacy of SARS-CoV vaccines. Our model systems provide a means for identifying the host factors that contribute to immune senescence and will allow us to evaluate whether changes in vaccine design or regimen will improve vaccine efficacy in senescent animals. The models provide clear rational to test candidate SARS vaccines in the hamster, ferret, and primate models in which pathology and clinical disease are more prominent following wild-type virus challenge. Our research provides a model for future experiments designed to characterize the components and inducers of the VRP-N–enhanced pulmonary inflammation, and suggests that vaccine regimens that contain N protein should be used with caution in human populations until further testing. The successful resurrection of a novel recombinant SARS challenge virus bearing zoonotic S glycoproteins suggests that it might be feasible to reconstruct other rare zoonotic SARS-CoVs that have never been successfully cultured, providing novel challenge viruses for vaccine and therapeutic drug testing against potential future zoonotic SARS introductions into human populations. Finally, these studies should encourage the development of senescent animal models of human disease and encourage vaccine testing and design against influenza, West Nile virus, and other pathogens that produce disproportionate disease burdens in the elderly [80,81].

## Supporting Information

Table S1 Titers and PRNT_80_ Dilutions for Individual Senescent Mice (146 KB DOC)

### Accession Numbers

The GenBank (http://www.ncbi.nlm.nih.gov/Genbank) accession number for the GD03-S glycoprotein sequence is AY525636.

## Abbreviations

ELISA: enzyme-linked immunosorbent assay
HAE: human airway epithelium
icGD03-S: recombinant chimeric virus encoding a synthetic S gene from the SARS-CoV strain, GD03
icSARS: recombinant Urbani strain of SARS-CoV
i.n.: intranasally
IU: infectious unit
N: nucleocapsid
OD: optical density
pfu: plaque-forming unit
PRNT: plaque reduction neutralization titer
RBD: receptor binding domain
S: spike
SARS-CoV: severe acute respiratory syndrome coronavirus
UNC: University of North Carolina
VRP: Venezuelan equine encephalitis virus replicon particle
VRP-HA: Venezuelan equine encephalitis virus replicon particle expressing the influenza A HA protein
VRP-N: Venezuelan equine encephalitis virus replicon particle expressing the Urbani SARS-CoV nucleocapsid
VRP-S: Venezuelan equine encephalitis virus replicon particle expressing the Urbani SARS-CoV spike
