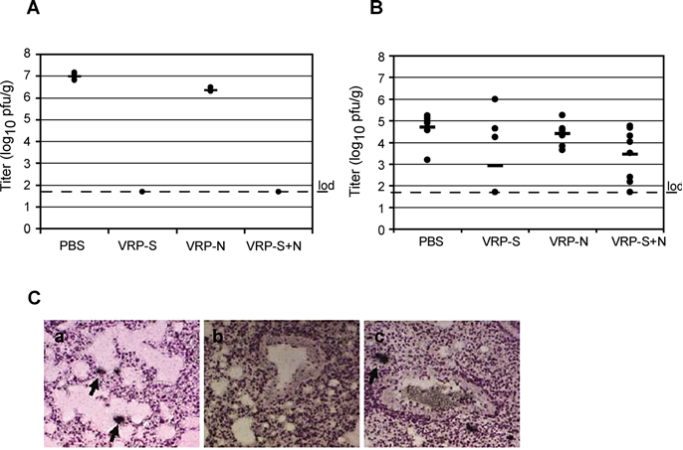# Correction: Vaccine Efficacy in Senescent Mice Challenged with Recombinant SARS-CoV Bearing Epidemic and Zoonotic Spike Variants

**DOI:** 10.1371/journal.pmed.0040080

**Published:** 2007-02-27

**Authors:** Damon Deming, Timothy Sheahan, Mark Heise, Boyd Yount, Nancy Davis, Amy Sims, Mehul Suthar, Jack Harkema, Alan Whitmore, Raymond Pickles, Ande West, Eric Donaldson, Kristopher Curtis, Robert Johnston, Ralph Baric

In *PLoS Medicine*, volume 3, issue 12: doi:10.1371/journal.pmed.0030525

Figure 4 part C was incorrect in the original article. The corrected Figure 4 is shown here.

## 

**Figure:**