# Supplementary material for: Vaccine Efficacy in Senescent Mice Challenged with Recombinant SARS-CoV Bearing Epidemic and Zoonotic Spike Variants
Source: PLoS Med. 2006 Dec 26;3(12):e525. doi: 10.1371/journal.pmed.0030525 (PMC1716185; doi:10.1371/journal.pmed.0030525)
Supplement: Supplementary file 1 [file pmed.0030525.st001.doc]

| **Experiment** | **Vaccine** | **Mouse** | **Challenge Virus** | **Lung Titer (log_10_pfu/g)** | **icSARS-CoV** | | **icGDO3-S** | |
| --- | --- | --- | --- | --- | --- | --- | --- | --- |
|  |  |  |  |  | **PRNT_80_ week 5** | **PRNT_80_ week 53** | **PRNT_80_ week 5** | **PRNT_80_ week 53** |
| **2** | VRP-S | 1 | icSARS | ND | 500 | 526 | <100 | <100 |
|  | VRP-S | 2 | icSARS | ND | 938 | 755 | <100 | <100 |
|  | VRP-S | 3 | icSARS | ND |  | 1417 |  | <100 |
|  | VRP-S | 4 | icSARS | ND | 585 | 159 | <100 | <100 |
|  | VRP-S | 5 | icSARS | ND | 695 | 493 |  | 114 |
|  | VRP-S | 6 | icSARS | ND | 1260 | 574 | <100 | 181 |
|  | VRP-S | 7 | icSARS | ND |  | 652 |  | <100 |
|  | VRP-S | 8 | icSARS | ND |  | 445 | <100 | <100 |
|  | VRP-N | 1 | icSARS | 6.1 |  |  |  |  |
|  | VRP-N | 2 | icSARS | 4.9 |  |  |  |  |
|  | VRP-N | 3 | icSARS | 5.2 |  |  |  |  |
|  | VRP-N | 4 | icSARS | 4.2 |  |  |  |  |
|  | VRP-N | 5 | icSARS | 5.8 |  |  |  |  |
|  | VRP-N | 6 | icSARS | 5.6 |  |  |  |  |
|  | VRP-N | 7 | icSARS | 5.4 |  |  |  |  |
|  | VRP-N | 8 | icSARS | 5.1 |  |  |  |  |
|  | VRP-S+N | 1 | icSARS | ND | >1600 | 549 | <100 | <100 |
|  | VRP-S+N | 2 | icSARS | ND | 628 | 150 | <100 | <100 |
|  | VRP-S+N | 3 | icSARS | ND | 1417 |  | 124 |  |
|  | VRP-S+N | 4 | icSARS | ND | 775 | 308 | <100 | <100 |
|  | VRP-S+N | 5 | icSARS | ND | 1159 | 346 | <100 | 107 |
|  | VRP-S+N | 6 | icSARS | ND |  | 594 | <100 | <100 |
|  | VRP-S+N | 7 | icSARS | ND | 1243 | 487 | <100 | <100 |
|  | VRP-S+N | 8 | icSARS | ND | 817 | 159 | <100 | <100 |
|  | VRP-HA | 1 | icSARS | 5.2 |  |  |  |  |
|  | VRP-HA | 2 | icSARS | 6.2 |  |  |  |  |
|  | VRP-HA | 3 | icSARS | 5.8 |  |  |  |  |
|  | VRP-HA | 4 | icSARS | 6.1 |  |  |  |  |
|  | VRP-HA | 5 | icSARS | 6.6 |  |  |  |  |
|  | VRP-HA | 6 | icSARS | 5.2 |  |  |  |  |
|  | VRP-HA | 7 | icSARS | 5.2 |  |  |  |  |

| **Experiment** | **Vaccine** | **Mouse** | **Challenge Virus** | **Lung Titer (log_10_pfu/g)** | **icSARS-CoV** | | **icGDO3-S** | |
| --- | --- | --- | --- | --- | --- | --- | --- | --- |
|  |  |  |  |  | **PRNT_80_ week 12** | **PRNT_80_ week 29** | **PRNT_80_ week 12** | **PRNT_80_ week 29** |
| **4** | VRP-S | 1 | icGDO3-S | ND | 237 | 100 | 100 | <100 |
|  | VRP-S | 2 | icGDO3-S | ND | 106 | 100 | <100 | <100 |
|  | VRP-S | 3 | icGDO3-S | 4.3 |  |  |  | <100 |
|  | VRP-S | 4 | icGDO3-S | ND |  |  |  | <100 |
|  | VRP-S | 5 | icGDO3-S | ND | 185 | 237 | 179 | <100 |
|  | VRP-S | 6 | icGDO3-S | 6.0 | 100 | 100 | <100 |  |
|  | VRP-S | 7 | icGDO3-S | ND | 291 | 212 |  |  |
|  | VRP-S | 8 | icGDO3-S | 4.7 | 100 | 100 | <100 |  |
|  | VRP-N | 1 | icGDO3-S | 3.6 |  |  |  |  |
|  | VRP-N | 2 | icGDO3-S | 4.6 |  |  |  |  |
|  | VRP-N | 3 | icGDO3-S | 4.5 |  |  |  |  |
|  | VRP-N | 4 | icGDO3-S | 5.3 |  |  |  |  |
|  | VRP-N | 5 | icGDO3-S | 3.8 |  |  |  |  |
|  | VRP-N | 6 | icGDO3-S | 4.3 |  |  |  |  |
|  | VRP-N | 7 | icGDO3-S | 4.6 |  |  |  |  |
|  | VRP-S+N | 1 | icGDO3-S | 4.7 |  |  |  | <100 |
|  | VRP-S+N | 2 | icGDO3-S | 4.0 |  | <100 |  | <100 |
|  | VRP-S+N | 3 | icGDO3-S | 2.2 | <100 | <100 | <100 | <100 |
|  | VRP-S+N | 4 | icGDO3-S | 4.3 | <100 | <100 | <100 | <100 |
|  | VRP-S+N | 5 | icGDO3-S | 2.4 | 114 | <100 | <100 | <100 |
|  | VRP-S+N | 6 | icGDO3-S | 4.8 | <100 | <100 | <100 |  |
|  | VRP-S+N | 7 | icGDO3-S | 3.5 | <100 |  | <100 |  |
|  | VRP-S+N | 8 | icGDO3-S | 1.7 |  |  |  |  |
|  | PBS | 1 | icGDO3-S | 4.9 |  |  |  |  |
|  | PBS | 2 | icGDO3-S | 5.0 |  |  |  |  |
|  | PBS | 3 | icGDO3-S | 4.7 |  |  |  |  |
|  | PBS | 4 | icGDO3-S | 4.6 |  |  |  |  |
|  | PBS | 5 | icGDO3-S | 5.1 |  |  |  |  |
|  | PBS | 6 | icGDO3-S | 4.9 |  |  |  |  |
|  | PBS | 7 | icGDO3-S | 5.2 |  |  |  |  |
|  | PBS | 8 | icGDO3-S | 3.2 |  |  |  |  |
